# The Beginning of an ECLS Center: First Successful ECPR in an Emergency Department in Romania—Case-Based Review

**DOI:** 10.3390/jcm12154922

**Published:** 2023-07-26

**Authors:** Paul Lucian Nedelea, Emilian Manolescu, Adi-Ionut Ciumanghel, Mihai Constantin, Alexandra Hauta, Oana Sirbu, Lidia Ionescu, Mihaela Blaj, Mihaela Corlade-Andrei, Victorita Sorodoc, Diana Cimpoesu

**Affiliations:** 1 Department of Emergency Medicine, “Grigore T. Popa” University of Medicine and Pharmacy, 700115 Iasi, Romania; 2 Emergency Department, “St. Spiridon” Emergency Clinical County Hospital, 700111 Iasi, Romania; 3 Anesthesia Intensive Care Unit, “St. Spiridon” Emergency Clinical County Hospital, 700111 Iasi, Romania; 4 2nd Internal Medicine Clinic, “St. Spiridon” Emergency Clinical County Hospital, 700111 Iasi, Romania; 5 Internal Medicine Department, “Grigore T. Popa” University of Medicine and Pharmacy, 700115 Iasi, Romania; 6 3rd Surgery Clinic, “St. Spiridon” Emergency Clinical County Hospital, 700111 Iasi, Romania; 7 S.C. Atto Digital USA S.R.L., 700045 Iasi, Romania

**Keywords:** emergency department, cardiac arrest, ECPR, extracorporeal membrane oxygenation, ECMO, ECLS, V-A ECMO, accidental hypothermia, cerebral performance category score

## Abstract

According to the latest international resuscitation guidelines, extracorporeal cardiopulmonary resuscitation (ECPR) involves the utilization of extracorporeal membrane oxygenation (ECMO) in specific patients experiencing cardiac arrest, and it can be considered in situations where standard cardiopulmonary resuscitation efforts fail if they have a potentially reversible underlying cause, among which we can also find hypothermia. In cases of cardiac arrest, both witnessed and unwitnessed, hypothermic patients have higher chances of survival and favorable neurological outcomes compared to normothermic patients. ECPR is a multifaceted procedure that requires a proficient team, specialized equipment, and comprehensive multidisciplinary support within a healthcare system. However, it also carries the risk of severe, life-threatening complications. With the increasing use of ECPR in recent years and the growing number of centers implementing this technique outside the intensive care units, significant uncertainties persist in both prehospital and emergency department (ED) settings. Proper organization is crucial for an ECPR program in emergency settings, especially given the challenges and complexities of these treatments, which were previously not commonly used in ED. Therefore, within a narrative review, we have incorporated the initial case of ECPR in an ED in Romania, featuring a successful resuscitation in the context of severe hypothermia (20 °C) and a favorable neurological outcome (CPC score of 1).

## 1. Introduction

Cardiac arrest (CA) is a significant public health issue, characterized by an incidence rate of 38–100 cases per 100,000 people in Europe and North America. The survival rate for patients who experience out-of-hospital cardiac arrest and receive CPR is around 10% worldwide [1,2,3]. To improve this survival rate, a new approach has been developed that combines traditional resuscitation techniques such as CPR and defibrillation with the use of extracorporeal membrane oxygenation (ECMO) to provide extracorporeal life support (ECLS). This combination of techniques is known as extracorporeal cardiopulmonary resuscitation (ECPR) and is used to save the lives of patients who do not respond to standard resuscitation methods.

Accidental hypothermia (AH) occurs when a person’s core body temperature drops involuntarily below 35 degrees Celsius [4,5,6,7,8]. Mild AH, which is a drop in temperature between 35 and 32 degrees Celsius, is common but does not typically cause major problems or require specialized care. However, moderate and severe or deep hypothermia, which is a drop in temperature below 28 degrees Celsius, is rare but can be very dangerous, even for healthy individuals; thus, it is considered a “low-incidence, high-impact” condition [4,5,9]. There have been reports of successful treatment for deep accidental hypothermia with excellent outcomes, including cases where the person’s core temperature reached a minimum of 13.7 degrees Celsius in adults and 11.8 degrees Celsius in children, accompanied by resuscitation durations as extensive as 8 h and 40 min [10,11,12,13,14].

In recent years, there have been significant improvements in the treatment of accidental hypothermia, particularly for patients with hypothermic cardiac arrest. Traditional methods of rewarming, such as dialysis and pleural lavage, were not very successful in saving these patients [6]. However, the use of extracorporeal life support has greatly increased the chances of survival for these patients. With new research and clinical experience, the treatment of accidental hypothermia has become more precise. ECLS has been particularly effective in improving survival rates and is now considered the preferred treatment option for hypothermic patients with unstable circulation or CA [6,15,16,17,18,19].

The aim of this article was to present, through a narrative literature review, the first case of ECPR being used to treat a patient with CA due to severe accidental hypothermia in an emergency department (ED) in Romania, as well as the development of the first ECPR program in Romania. The case will be discussed within the context of related literature.

## 2. Case Presentation

A presumed 42-year-old homeless male was found unconscious in a canal in winter and was admitted to the ED of the “Sf. Spiridon” Emergency County Hospital in Iasi on 12 December 2022 with severe hypothermia. The patient’s primary medical history involved chronic alcohol abuse. Upon clinical examination, the patient displayed profound hypotension (80/42 mmHg), bradycardia (25 bpm), bradypnea (5 bpm), and coma with a Glasgow Coma Scale score of 5 (E2, V2, and M1). Arterial blood gas analysis indicated severe metabolic acidosis with a pH of 7.01, PaCO_2_ of 20 mmHg, and PaO_2_ of 68 mmHg. Lactic acidosis was present with a value of 5 mmol/L alongside moderate anemia (Hb of 8.7 g/L) and moderate hypokalemia (K 2.8 mmol/L). The patient’s admission ethanol level was 3.7 g/L. Liver cytolysis or cholestasis were not identified, and renal function was normal. The core temperature measured at the tympanic level was 20 °C. Passive and active warming measures were initiated, including the use of a forced-air warming blanket and warmed fluid infusions (2000 mL Ringer’s lactate). A dosage of 40 mmol of potassium chloride 7.45% was administered to assist in correcting hypokalemia. Bag and mask ventilation was used initially, and orotracheal intubation was performed.

Fifty minutes following admission to the ED, the patient developed CA. Traditional cardio-pulmonary resuscitation (CPR), which includes mechanical chest compressions (AutoPulse device), was immediately initiated, while rewarming procedures were concurrently continued. The decision to begin ECPR was made at the onset of CPR maneuvers.

A veno-arterial extracorporeal membrane oxygenation (V-A ECMO) system was installed and started 50 min after the onset of CPR maneuvers with a flow of 2.5 L/min/m^2^, FiO_2_ 100% using a right femoral-femoral approach (a 23 F venous catheter and a 17 F arterial catheter were used). Initially, during cannulation of the left common femoral vein, a complication arose, as the catheter could not be fully inserted; thus, the decision was made to place the catheter in the right side where the arterial catheter was already positioned. At the puncture site of the left common femoral vein, mechanical compression was applied for 3 h. Subsequent Doppler ultrasound revealed normal blood flow in the left femoral artery and vein, as well as the absence of a local hematoma. On the right side, it showed absence of blood flow in the distal femoral artery, so a 6 Fr distal leg perfusion catheter was inserted with echo-guidance 2 h after ECLS was started, resulting in improvement of blood flow post procedure. 

The total time taken to rewarm the patient from a temperature of 20 °C to 35 °C was 5 h, at a speed of 3 °C per hour. When the patient’s core temperature reaches 29 °C, ventricular fibrillation (VF) ensues and is successfully converted to sinus rhythm after two defibrillation attempts of 200 J each. Sedation, vasopressors, and anticoagulation with unfractionated heparin were started before the patient was transferred to the intensive care unit (ICU). A cystocath was inserted, as a urethral catheter could not be placed.

Upon admission to the ICU, the patient was mechanically ventilated with an FiO_2_ of 0.5, a VT of 420 mL, RR of 18, and PEEP of 5. He was sedated with 100 mg/h of propofol and had a central temperature of 35 °C. The patient’s ECMO parameters were a flow of 1.8 L/min/m^2^, FiO_2_ of 1, gas flow of 3 L/min, and temperature at 35 °C. 

The patient’s pH was 7.29, with PaCO_2_ at 37 mmHg, PaO_2_ at 426 mmHg, lactate at 6.5 mmol/L, and BE at −8.4 mmol/L. The FiO_2_ was decreased to 0.4 to maintain SpO_2_ between 92–98% and PaO_2_ between 80–150 mmHg. On admission, the patient’s blood pressure was 98/56 (66) mmHg, and the heart rate was 92 bpm with norepinephrine at 0.5 µg/kg/min.

Laboratory tests revealed severe anemia, with a drop in hemoglobin from 8.7 g/L to a level of 5.7 g/dL. The patient also had thrombocytopenia at 112.000/mL and leukocytosis at 11.250/mm^3^. Coagulation disorders were present, including an INR of 1.69, prothrombin activity at 49%, PTTa of 55.7 s, PT of 18.8 s, and moderate liver cytolysis. The patient also had rhabdomyolysis, hyperglycemia, and hypoalbuminemia. Electrolyte levels were normal. To keep hemoglobin levels above 7 g/dL, blood transfusions were given in both the ED (2 units of red blood cell concentrate and 2 units of fresh frozen plasma) and the intensive care unit (5 units of red blood cell concentrate). Unfractionated heparin was given to maintain a PTTa between 50–70 s, and the patient’s hemodynamic support was gradually reduced. Early initiation of enteral nutrition occurred within 24 h of admission to the ICU, and subsequent increments were made gradually.

Upon 48 h after admission to the ICU, the patient’s left ventricular ejection fraction (LVEF) was 55%, with grade II mitral regurgitation and a RV-RA gradient of 30 mmHg without any other changes. Given his good cardiac function, the decision to wean off ECMO was made on the second day, and on the third day, the ECLS support was removed in the operating room by the vascular surgical team simultaneously with a cystostomy to investigate a large hematoma that had developed in the bladder. A specific source of the bleeding in the bladder was not found. The patient’s post-operative course was favorable, with the resolution of the bladder hematoma and no complications at the ECLS cannula insertion sites. 

The patient’s first neurological examination, performed without sedation 48 h after admission, revealed generalized myoclonic seizures. Symptomatic treatment was achieved with carbamazepine after a CT scan ruled out an intracranial lesion. The patient stopped receiving sedatives on day 4 and was extubated the same day of the ICU stay and transferred to the medical ward on day 9 with a Glasgow-Pittsburgh Cerebral Performance Category (CPC) score indicating a level 2. 

On the medical ward, the patient’s clinical condition was characterized by slightly altered neurological function, being conscious, cooperative, disoriented in time and space, and slightly verbally aggressive. On day 10, in the context of a psychomotor agitation episode with a tendency to suppress catheters, mechanical restraint was used for 24 h. Laboratory tests showed a normochromic normocytic anemia, leukocytosis, thrombocytosis, an inflammatory syndrome with inflammatory markers (CRP 22.16 mg/dL, ferritin >500 ng/mL, iron 8 microg/dL), INR 1.65, hyperfibrinogenemia, GGT 506 U/L, lactate 27.2 mg/dL, hypoproteinemia with hypoalbuminemia, and a deficit in vitamin B12 and folic acid. Thyroid function and viral markers were within normal range. Positive blood culture results were identified for *Enterococcus faecium*, as well as in the urine culture. The tracheal aspiration culture was positive for *Acinetobacter baumannii* and gram-negative bacilli and negative for fungi. Cultures taken from secretions on a right leg wound revealed the presence of *Stenotrophomonas maltophilia*, but no fungi were detected. Cultures taken from a left leg wound identified *Acinetobacter baumannii* and *Klebsiella pneumoniae*, and again, no fungi were present. A urine catheter tip culture was negative. The initial antibiotic treatment, which included polymyxin E and cefepime, was changed to a regimen of polymyxin E, imipenem/cilastatin, and linezolid. This led to a resolution of inflammatory syndrome on day 26.

The neurological progress was favorable, with symptoms mainly consisting of toxic and nutritional polyneuropathy, with a CPC score of 1. Given the patient’s social status and lack of housing, he was discharged and placed in a social center, where he is receiving ongoing psychological and social support. 

## 3. Discussion

The European Resuscitation Council (ERC) Guidelines 2021 recommend the use of extracorporeal membrane oxygenation (ECMO) in certain specific cases of cardiopulmonary resuscitation (CPR). Thus, patients in cardiac arrest who can potentially benefit from the utilization of ECMO are adult patients experiencing refractory cardiac arrest who have failed to respond to standard CPR and advanced life support measures and have a potentially reversible underlying cause. It should be used as a rescue therapy and be considered as part of a structured and coordinated system of care that includes an experienced ECMO team and an appropriate referral center. Hence, the decision should be assessed and determined individually for each case, considering the patient’s underlying condition, the availability of ECMO resources, and the patient’s chances of survival and recovery [17]. Starting from the desire to implement an ECPR system in Romania at the University of Medicine and Pharmacy (UMF) “Grigore T. Popa” in Iasi, we have developed a multidisciplinary project to equip our center with an ECLS device: “Multidisciplinary Research and Development Medical Platform in the North-East Region” (CENEMED) of the UMF “Grigore T. Popa”. Our ECMO center was established without the support of a national coordinated system, despite sporadic use in some ICUs. Thanks to the CENEMED project, we were able to acquire a Cardiohelp (Maquet) ECMO machine and necessary ECLS consumables. However, due to limited institutional commitment, ECPR procedures are not currently funded by the Romanian National Health Fund, and we rely on the consumables purchased through the project. The development of our ECMO center occurred in four main stages: initialization, training, preparation, and activation.

Initialization. The idea of initiating an ECMO/ECPR center in our facility emerged in 2018, but due to the impossibility of obtaining funding from the Ministry of Health, we had to apply, through the university, for a European grant. Thus, 4 years later, the CENEMED project was realized, through which we managed to equip the center with an ECMO machine and the related consumables.

Training. From 26 to 30 September 2022, a team of five doctors from the emergency department of the “Sf. Spiridon” Emergency County Hospital in Iasi participated in an ECMO training course in Regensburg, Germany. Local training was conducted with the guidance of specialists from Getinge, aimed at building our confidence in managing the ECPR technique. This included training on both priming and cannulation. Following this, our team engaged in internal training sessions to discuss and create an action plan for implementing ECPR in cases of cardiac arrest.

Preparation. Multiple internal protocols were integrated into everyday practice, encompassing mechanical ventilation strategies, patient weaning plans, anticoagulation protocols, and pharmacotherapy adjustments. Moreover, specific protocols were developed and rehearsed to handle critical scenarios during ECMO, such as oxygenator dysfunction, accidental decannulation, extracorporeal system failure, and recirculation. Specific criteria were established to identify potential candidates for initiating a “Code ECPR”. These criteria include the following: (1) age between 18 and 65, (2) witnessed cardiac arrest, (3) duration of no flow (time from collapse to CPR initiation, including bystander efforts) less than 10 min, (4) resuscitation efforts exceeding 10 min without achieving return of spontaneous circulation (ROSC), (5) emergency medical service (EMS) transportation to the hospital within 20 min, and (6) absence of patient-related factors that would hinder ongoing resuscitation (trauma, palliative care involvement, or metastatic cancer). All cases requiring ECPR are managed by a well-trained team of five emergency physicians and two intensivists who are fully engaged in the activities of the ECMO center. Only vascular surgeons and cardiologists may be called if needed, and no other health professionals such as perfusionists, cardiovascular surgeons, respiratory therapists, etc. are involved in the ECLS therapy.

**Figure 1:**
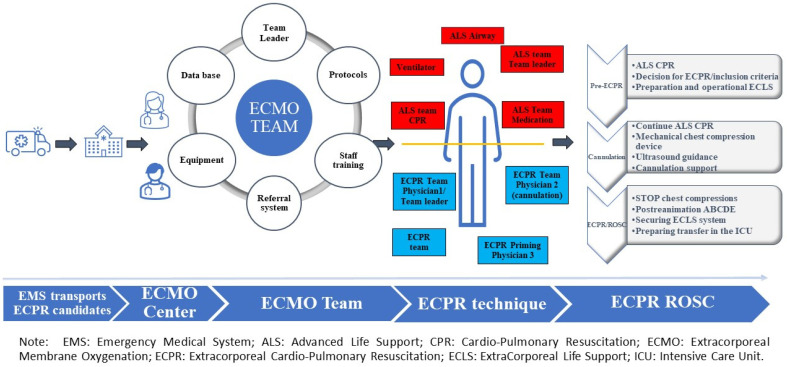
Stages of the ECPR process in our center.

Activation. The ED-ECPR program has been designed to ensure the presence of an ECMO team physician during each shift. This physician is capable of promptly identifying cases that require ECPR and, after consulting with the team leader, initiating the priming of the device and cannulation procedure. Other team members, who are required to arrive at the hospital within 30 min of code ECPR activation, provide additional support throughout the process (Figure 1).

Although ECMO has been used in Romania since 2011 in eight intensive care units of university hospitals, its use has been limited to cases of cardiogenic shock (V-A ECMO) or ARDS (V-V ECMO) [20]. Although our initial plan was to become familiar with the V-V ECMO cannulation technique, we had an opportunity a month before the case we are presenting to attempt ECPR on a patient with cardiorespiratory arrest caused by a massive myocardial infarction. Unfortunately, this attempt was unsuccessful, and the patient passed away a few days later. Due to a shortage of consumables at that time, the decision was made to continue only with patients in cardiac arrest.

To the best of our knowledge, the case we are now reporting is the first documented case in Romania of a patient in cardiorespiratory arrest who underwent ECLS (ECPR) in the ED and achieved a good CPC score. ECPR and ECLS rewarming, which is made possible by recent advancements in technology such as miniaturization and improved efficacy, safety, and transportability of ECLS devices, may lead to higher survival rates (40–100% vs. 20–40%) and better neurological outcomes compared to standard CPR alone in patients suffering from CA due to severe accidental hypothermia [6,17,18,19,21]. ECPR in hypothermic patients can achieve positive neurological outcomes despite prolonged or diminished blood flow. However, it should be avoided if core temperature exceeds 30 °C, if severe trauma is present, or if recovery would result in diminished quality of life [21]. V-A ECMO is the preferred method of ECPR because it requires less anticoagulation and can provide circulatory and respiratory support beyond ROSC [22,23,24]. Patients with witnessed hypothermic cardiac arrest have higher survival rates compared to those with unwitnessed cases, with avalanche survivors having the lowest likelihood. Factors linked to reduced survival include male gender, elevated initial body temperature, low pH, and high serum potassium [25,26].

The utilization of ECMO during ECPR poses unique challenges. Unlike severe cardiogenic shock, which typically occurs in specific settings such as the catheterization laboratory, ICU, or operating room, cardiac arrest can occur unpredictably anywhere within a hospital, including the ED, where ECPR programs are increasingly developing. It is highly advisable to establish a strong connection between ECPR programs and hospital intensive care units that possess expertise in managing patients on ECMO. Additionally, patients should be quickly transferred to a referral center for proper management if possible [27,28].

Survival rates with ECPR are highly variable due to delayed decision making, often after 30–40 min of unsuccessful standard CPR. The duration of standard CPR is an independent prognostic parameter for refractory out-of-hospital cardiac arrest (OHCA). Consequently, as the duration of standard CPR increases, the outcome worsens, and this period during cardiac arrest is referred to as the low-flow phase [29]. To optimize outcomes, in the case of eligible patients who do not respond to the initial 10 min of standard CPR, it is important to anticipate and have ECPR readily accessible and initiate it within a frame of 20 to 60 min. This is crucial to minimize the period of low flow and increase the chances of survival [30,31,32,33]. Patients who experience a low flow period exceeding 90 min are unlikely to derive significant benefits from ECPR [29]. Present guidelines emphasize that the timeliness and quality of chest compressions significantly impact the efficacy of all subsequent procedures [34]. Consequently, initiating CPR immediately after the collapse to minimize the duration of no-flow becomes essential. Furthermore, ensuring high-quality CPR during the low-flow period is equally imperative [35]. For the purpose of achieving optimal outcomes, the monitoring of expired carbon dioxide (EtCO_2_) is recommended, as it is a validated indicator of survival in cardiac arrest. A low EtCO_2_ value of <10 mmHg has been linked to a lower survival rate [36].

The initiation of ECPR necessitates a team that is specifically trained and well-coordinated. Additionally, during the cannulation process, a dedicated team leader should oversee the resuscitation procedure. The choice to implement ECMO in the case of CA must be made promptly and ideally in regional referral centers [27]. In the case we report, the decision to initiate ECPR was made promptly after CA occurred. A team trained for the procedure was already in place, resulting in initiation of ECLS at 50 min from the onset of CA. In our case, the duration between the occurrence of cardiac arrest and the initiation of ECPR was comparable to that found in a retrospective analysis of an observational multicenter cohort study conducted in Japan [37]. According to the study, patients who achieved successful resuscitation and had a CPC score level of 1 or 2 had a median low-flow time of 55 min for ECPR (with an interquartile range of 45–66 min). 

In our patient, CPR included from the beginning the application of a mechanical chest compression device (AutoPulse Resuscitation System) to ensure high quality chest compressions. According to data from The International Hypothermia Registry (IHR), mechanical devices for CPR were utilized in 39% of cases involving hypothermic patients [19]. In situations where high-quality manual chest compressions are impractical or unsafe, mechanical chest compressors operated by trained medical professionals serve as a viable alternative [35]. 

Placing ECMO during cardiac arrest is complicated and requires specialized knowledge. The cannulation for ECMO can be done either through a percutaneous method using vessel puncture and sequential dilations guided by ultrasound following the Seldinger technique or a direct surgical approach to the femoral artery [38]. In our case, the cannulation method was percutaneous, as it was the most accessible and fastest option for which we were prepared. In a retrospective study conducted at a university hospital with a significant number of ECMO implantations, it was discovered that among the 814 patients who underwent implantation (485 surgical and 329 percutaneous), the percutaneous approach was associated with a lower incidence of local infections (16.5% vs. 27.8%, *p* = 0.001), comparable rates of limb ischemia (8.6% vs. 12.4%, *p* = 0.347), similar occurrences of neurological complications (2.6% vs. 2.3%, *p* = 0.779), and a higher 30-day survival rate (63.8% vs. 56.3%, *p* = 0.034). However, the percutaneous method was linked to a higher incidence of post-decannulation vascular complications (14.7% vs. 3.4%, *p* < 0.001), primarily local bleeding necessitating surgical intervention (9.4% vs. 1.5%, *p* < 0.001) [39]. Irrespective of the cannulation technique employed, echocardiography plays a vital role in verifying the accurate positioning of guides and cannulas prior to the installation of ECMO [40].

The size of the cannulas plays a critical role in the success of ECPR. We chose a 23 Fr for the venous cannula and a 17 Fr for the arterial cannula. Proper selection of the diameter of the cannulas is necessary to ensure efficient blood drainage from the patient as well as to provide satisfactory blood reinjection to the patient. Although there is a lack of evidence regarding the ideal ECMO flow rate required to maintain optimal organ perfusion, it is recommended to utilize a minimum of 23–25 Fr for the drainage cannula and 17–19 Fr for the reinjection cannula in adults [38].

One of the complications that arose was represented by the ischemia of the lower limb on which the arterial cannula was placed. To prevent the blockage of the femoral artery and lack of blood flow to the leg caused by an arterial cannula, it is recommended to also place a cannula for reperfusion within the superficial femoral artery of the same leg. By connecting the reperfusion cannula to the arterial circuit, it enables sufficient perfusion of the distal portion of the cannulated lower limb. It is best to place this cannula early, and it can be inserted either surgically or using ultrasound guidance [41]. In our case, the Doppler ultrasound showed a lack of distal flow at the site of arterial cannula placement, so 2 h after initiating ECMO, we placed a distal-leg 6 Fr reperfusion catheter with a good flow monitored by an hourly Doppler. This 2 h delay was caused by the unfortunate lack of connectors between the distal leg perfusion catheter and the arterial circuit, which were eventually obtained from another hospital’s cardio-vascular surgery department.

Continuous ECPR should be administered to patients experiencing hypothermic cardiac arrest until the return of spontaneous circulation (ROSC) is achieved. In our case, ROSC was immediately achieved after initiating the ECMO device. Two episodes of VF were resolved by defibrillation when the patient’s core temperature reached 29 degrees Celsius. The decision to persist with care in the absence of immediate substantial improvement poses a challenging dilemma [42]. The in-hospital HOPE (Hypothermia Outcome Prediction after Extracorporeal Life Support) score has greatly enhanced outcome prediction for hypothermic patients experiencing cardiac arrest and undergoing ECLS rewarming [43]. In our case, the HOPE survival probability was 93%. 

Reliable predictors of outcome or efficacy for hypothermic patients in cardiac arrest are currently lacking [15,16,17]. The serum potassium level is presently the only widely accepted prognostic indicator for hypothermic patients experiencing cardiac arrest. In the case of hypothermic avalanche victims, a cutoff value of 8 mmol/L is considered futile for attempted resuscitation, while for all other victims, the cutoff is 12 mmol/L [44,45]. Other predictors have also been identified and described, including the absence of asphyxia, lower initial serum lactate levels, higher pH levels, and younger age, all of which have been associated with improved neurological outcomes in survivors of hypothermic out-of-hospital cardiac arrest treated with ECPR [22,46,47]. The findings from the IHR retrospective cohort study [19] supported these results, identifying factors associated with improved survival. These factors included witnessing the cardiac arrest (71% vs. 30%; *p* < 0.005), achieving ROSC before rewarming (35% vs. 7%; *p* < 0.007), lower potassium levels (3.5 ± 0.9 vs. 6.5 ± 2.5 mmol/L, *p* = 0.001), and lower lactate levels (11 ± 5 vs. 16 ± 5 mmol/L, *p* = 0.003) at the time of hospital admission. Additionally, patients with asphyxia showed a tendency towards higher mortality (49% vs. 23%, *p* < 0.059). Survivors and non-survivors did not exhibit any differences in underlying cardiac rhythm, core temperature, or pH value at the time of hospital admission. In our case, moderate hypokalemia (K 2.8 mmol/L) was identified and corrected by using warmed fluid infusions (2000 mL Ringer’s lactate) and a dosage of 40 mmol of potassium chloride 7.45%.

The total time taken to rewarm the patient from a temperature of 20 °C to 35 °C was 5 h, at a rate of 3 °C per hour. The speed at which hypothermic patients are rewarmed depends on the technique used. Active external rewarming is known to gradually increase core temperature at a rate of 0.1 to 3 °C per hour. On the other hand, peritoneal lavage, thoracic lavage, and hemodialysis have the potential to elevate core temperature at rates of 1–3 °C/h, 3 °C/h, and 2–4 °C/h, respectively [48]. Moreover, ECMO has the capability to raise core temperature at a rate of 4 °C per hour [49]. The literature lacks a well-defined consensus on the optimal speed of rewarming. One suggested approach is to limit the maximum speed to 4 °C per hour [50] while ensuring that the temperature difference between the patient and ECMO does not exceed 10 °C [51]. In a pig model study, two ECMO flow rates (1.5 L/min vs. 3 L/min) and two temperature goals (5 degrees Celsius above body temperature vs. 38 degrees Celsius) were compared. The group with a flow rate of 3 L/min showed improved cardiac output [51].

Post-arrest care following cannulation is a crucial element of a successful ECPR program, as emphasized in both the ARREST and Prague OHCA trials [52,53]. After ROSC has occurred, local protocols should be followed when implementing targeted temperature management (TTM), and efforts should be made to prevent post-resuscitation hyperthermia [24], this being easily achieved through the extracorporeal circuit. The precise timing of ECPR and the ideal target temperature following ECPR remain unknown at present [54]. If the target core temperature is reached but there is no return of spontaneous circulation (ROSC), it is advisable to consider discontinuing ECPR. The decision to discontinue treatment may also rely on additional clinical factors, including uncontrollable bleeding, further insights into the underlying cause of cardiac arrest, or indications of significant brain injury due to oxygen deprivation [6,55]. Moreover, brain death frequently occurs as a cause of death after ECPR, and in such cases, the option of organ donation should be taken into consideration [23,56].

After ECPR, management focuses on preserving adequate organ perfusion by restoring the cardiac output of the patient. Once extracorporeal circulation is established, chest compressions can be stopped. The effectiveness of defibrillation is enhanced when there is an improvement in coronary perfusion pressure and sufficient oxygen supply from the extracorporeal pump. 

Managing hyperoxia after ECPR is challenging, and oxygen supply must be calibrated to avoid negative effects on neurological and cardiovascular outcomes. ECMO has the ability to significantly elevate arterial partial pressures of oxygen (PaO_2_) and reduce carbon dioxide (PaCO_2_). However, there is limited information available regarding the optimal oxygenation targets and the safest levels and rates of change in PaCO_2_. It has been observed that drastic decreases in PaCO_2_ (<30 mmHg) within the initial 48 h of ECMO and rapid fluctuations in PaCO_2_ from pre to post cannulation are associated with neurological complications [57,58]. There is a need for prospective studies to assist in determining the ideal gas exchange targets following the initiation of ECPR.

It is recommended to maintain the mean arterial pressure within the range of 65 to 75 mmHg, often with the use of vasopressors. Invasive blood pressure monitoring is necessary, and the right radial artery should be catheterized to detect the potential of Harlequin syndrome and hypoxemia of pulmonary origin. Furthermore, aggressive volume resuscitation may be necessary to ensure sufficient preload for the support of ECPR [22].

Appropriate anticoagulation is of paramount importance in the care of post-ECPR patients. It helps prevent thrombotic complications, preserves ECMO function and maintains the delicate balance between hemostasis and bleeding. There is limited evidence indicating potential benefits for patients when utilizing a lower target anticoagulant dose [59]. Close monitoring of coagulation parameters and individualized anticoagulation strategies are necessary to optimize patient outcomes. The use of anticoagulants such as unfractionated heparin or direct thrombin inhibitors reduces the risk of circuit clotting, thromboembolic events, and subsequent organ dysfunction. Monitoring anticoagulation parameters, such as activated clotting time (ACT) or anti-Xa levels, is crucial to ensure effective anticoagulation while minimizing bleeding complications. In our case, despite using a lower anticoagulation dose of unfractionated heparin, no ischemic events occurred during the patient stay in ICU. However, a bladder bleeding hematoma, likely related to the suprapubic catheterization associated with the anticoagulation, occurred during the course of treatment. Fortunately, this complication was effectively resolved.

The recognition of medical nutrition’s importance in the management of ECPR/ECLS patients is growing, as it is increasingly acknowledged as a critical factor that contributes to favorable outcomes in individuals experiencing a state of severe catabolism [60,61]. During the patient’s stay in the ICU, he remained hemodynamically stable with good oxygenation, allowing us to administer isocaloric enteral nutrition. Enteral nutrition was initiated early, at 24 h after admission to the ICU, and gradually increased. Digestive tolerance was good, enabling a gradual increase in the volume of enteral solutions administered, reaching the required caloric intake by day 5 of intensive care. Following weaning off mechanical ventilation, the patient was fed with a hypercaloric enteral regimen.

ECPR demands specialized equipment and personnel, making it a costly intervention. The assessment of its cost-effectiveness and whether the expenses justify the incremental improvements in survival compared to standard CPR will depend on factors such as the hospital, country, and region. Accurate estimation of ECPR effectiveness is crucial, highlighting the importance of conducting cost-effectiveness analyses in future large randomized controlled trials. It is worth mentioning that existing economic assessments focus on the cost per patient in centers that already have established ECMO programs. However, these assessments do not account for the expenses involved in initiating a new ECMO program, let alone implementing a new ECPR program in a center where extracorporeal cardiopulmonary support was previously unavailable. These start-up costs can render the overall cost per ECPR patient unaffordable, particularly given the rarity of suitable ECPR candidates [62,63,64,65]. Beyond economic aspects, what stands out as important is identifying evidence that leads to a standardization of ECPR approach, clear patient selection criteria, and appropriate timing [43,65,66] for its use in the emergency department [67,68,69] or in the prehospital setting [52,53,70,71] 

## 4. Conclusions

Accidental hypothermia, compared to other conditions that can lead to cardiac arrest, is rare. Although ECPR is a recommended procedure to be used in cases of cardiac arrest in hypothermic patients, even at large medical centers, it is rare to treat more than 20 patients per year. To date, the most extensive research on the topic has only included a few hundred patients with only two trials with inconclusive results. Combining data from multiple studies is crucial for advancing methods for diagnosing, treating, and predicting outcomes for patients with ECPR. We presented a case of successful resuscitation after severe hypothermia (20 °C) with a favorable neurologic outcome despite a few complications appearing during the ECPR/ECLS. This successful case of ECPR represents a significant milestone for Romania, as ECPR had not been previously used in the country, including in intensive care or other emergency departments. This encourages us to continue our work and contribute further to the research in this field.

ECPR is a highly specialized and invasive procedure and should only be performed in specialized centers with the appropriate equipment and trained personnel. Clear communication and defined processes are crucial to ensure the best possible outcome for patients undergoing ECPR. Effective decision making, prompt timing, precise execution, appropriate intensive care, and thorough follow-up are vital components in increasing the success rate of ECPR, for optimizing outcomes and ensuring the best chance of recovery in patients with hypothermia who have experienced cardiac arrest. A multidisciplinary approach including more often the input of emergency medicine together with anesthesia and critical care and other specialties’ teams is important to ensure that the patient receives the best possible care.

## Data Availability

The data that support the findings of this work are available on request from the corresponding author.
